# Selection of an Insurance Company in Agriculture through Hybrid Multi-Criteria Decision-Making

**DOI:** 10.3390/e25060959

**Published:** 2023-06-20

**Authors:** Adis Puška, Marija Lukić, Darko Božanić, Miroslav Nedeljković, Ibrahim M. Hezam

**Affiliations:** 1 Government of Brčko District of Bosnia and Herzegovina, Department of Public Safety, Bulevara Mira 1, 76100 Brčko, Bosnia and Herzegovina; adispuska@yahoo.com; 2 Faculty of Business Economics and Entrepreneurship, Economics, Finance and Banking, Mitropolita Petra 8, 11000 Belgrade, Serbia; lukicmmarija@gmail.com; 3 Military Academy, University of Defence in Belgrade, Veljka Lukica Kurjaka 33, 11000 Belgrade, Serbia; 4 Institute of Agricultural Economics, Volgina 15, 11060 Belgrade, Serbia; miroslavnedeljkovic2015@gmail.com; 5 Statistics & Operations Research Department, College of Sciences, King Saud University, Riyadh 11451, Saudi Arabia; ialmishnanah@ksu.edu.sa

**Keywords:** crop insurance, insurance companies, fuzzy number, LMAW, entropy, CRADIS, agricultural production

## Abstract

Crop insurance is used to reduce risk in agriculture. This research is focused on selecting an insurance company that provides the best policy conditions for crop insurance. A total of five insurance companies that provide crop insurance services in the Republic of Serbia were selected. To choose the insurance company that provides the best policy conditions for farmers, expert opinions were solicited. In addition, fuzzy methods were used to assess the weights of the various criteria and to evaluate insurance companies. The weight of each criterion was determined using a combined approach based on fuzzy LMAW (the logarithm methodology of additive weights) and entropy methods. Fuzzy LMAW was used to determine the weights subjectively through expert ratings, while fuzzy entropy was used to determine the weights objectively. The results of these methods showed that the price criterion received the highest weight. The selection of the insurance company was made using the fuzzy CRADIS (compromise ranking of alternatives, from distance to ideal solution) method. The results of this method showed that the insurance company DDOR offers the best conditions for crop insurance for farmers. These results were confirmed by a validation of the results and sensitivity analysis. Based on all of this, it was shown that fuzzy methods can be used in the selection of insurance companies.

## 1. Introduction

Agricultural production is a risky business due to the long production cycle and the vulnerability of crops to adverse weather conditions, pests, diseases, and other disasters that affect agricultural yields [1]. As a result of these influences, uncertainty is created in the business of farmers, resulting in reduced profitability and diminished inflows of funds, which affects their business. To reduce uncertainty in agricultural production, farmers choose different risk and uncertainty management strategies, including crop insurance [2]. Farmers insure their crops with insurance institutions. Insurance is a risk management form used to limit potential losses. Crop insurance is an effective mechanism that, in addition to dispersing risk, helps to promote green agriculture [3].

Crop insurance helps to stabilize farmers’ income and provides better financial results for farms. It helps farmers to stabilize agricultural production and also increase their income at the same time [4]. With crop insurance, a contract is signed that protects the farmer in the event of natural disasters and damage to crops [5]. Therefore, crop insurance is the main driver of changes in agriculture, as it leads to increased productivity by protecting farmers against possible risks [6]. When farmers insure their crops, they may be prepared to take on more risky agricultural practices that can have serious economic, social, and ecological effects on agriculture [7]. This can also be the negative side of crop insurance, as it relieves farmers of risk and leads to increased production and the planting of only high-income crops. This has led to an expansion in agriculture, resulting in deforestation, water degradation, and significant greenhouse gas emissions [8].

Therefore, sustainable agricultural production should be applied, and crop insurance should only be a measure that supports such production [9]. It is necessary for governments to engage in programs that encourage farmers to insure their crops while applying the principles of sustainable agricultural production. Therefore, it is necessary to improve the overall productivity of sustainable production to reduce the impact of agriculture on the environment [2]. Crop insurance is a means to reduce economic losses and protect production, where the farmer will reduce agrochemical inputs, thereby mitigating the impact on the environment and promoting sustainable, or green, agricultural production [3]. Insurance in agribusiness is a commercial activity and countries around the world have taken an active role in this area, with a clear interest and the goal of maintaining the overall productivity of the national economy and promoting rural development. Crop insurance has become an important tool for managing agricultural risks worldwide [10].

The research was conducted from the client’s perspective and aims to protect the interests of farmers in agricultural production, as crop insurance plays a significant role in this endeavor. This paper will select an insurance company in order to choose the most appropriate insurance for farmers in the Republic of Serbia. Therefore, this study examined five insurance companies that provide crop insurance services. These insurance companies will be evaluated based on nine criteria. Based on this examination, it can be seen that there is a problem regarding decision-making, where multiple alternatives are valued using multiple criteria. This problem is solved by using multi-criteria decision-making (MCDM). In order to choose the insurance that best secures crops, a combination of methods for objectively and subjectively determining the weights of criteria will be used, and then these insurance policies will be ranked. Based on this, the aim of this research is to use an approach that reduces subjectivity in the selection of crop insurance. This approach contributes to making a realistic decision that is not burdened by the subjectivity of the decision-maker. In this case, the choice of insurance represents a decision-making problem. This combination of methods for objectively and subjectively determining the weighting will contribute to the safety of choosing an insurance company, as it will reduce subjectivity in decision-making. In addition, expert decision-making will be used, whereby experts will assess the policies using linguistic values. This research was conducted with five experts, including three experts who are owners of large agricultural estates in the Republic of Serbia, and two experts who are professors at the Faculty of Agriculture, specifically in the Department of Agricultural Economics. In this way, practice and theory are connected in the selection of crop insurance. To process these values, a fuzzy approach to decision-making will be used.

To determine these weights, the fuzzy entropy method and fuzzy LMAW (the logarithm methodology of additive weights) method will be used, which will determine the various weights based on expert subjective evaluations. After that, the fuzzy CRADIS method (the compromise ranking of alternatives from distance to an ideal solution) will be used to rank the insurance companies. By applying these methods, a hybrid methodology is developed to select the insurance company with the best indicators. Based on all of this, the research goal is set: to use fuzzy MCDM methods to select an insurance company that provides the best crop insurance, using the example of the Republic of Serbia. The contribution of this research is reflected in the following:

- The application of fuzzy MCDM methods for selecting an insurance company that provides the best conditions for crop insurance;
- Development of a methodology that will reduce subjectivity in decision-making;
- Advancement of agricultural production through the application of crop insurance in the Republic of Serbia.

In addition to the introduction, this paper is divided into five more sections. The literature review in Section 2 presents previously published papers that have used MCDM methods to assess insurance. The third section deals with defining the research problem with the definition of criteria and alternatives. The fourth section explains how the research was conducted, and which methods are used in this research. Section 5 discusses the results of determining criteria weights and ranking insurance companies. This section validates the research results and sensitivity analysis. Section 6 discusses and presents the research results in more detail. The conclusion provides the most important results and the limitations of this study, as well as guidelines for future research.

## 2. Literature Review

It is very important in agricultural production to increase the productivity of farms. This increase in productivity is achieved through crop insurance. In order for farmers to use crop insurance more fully, governments provide subsidies to farmers. Yu and Sumner [11] have shown that subsidies encourage farmers to buy crop insurance, which increases the expected return on insured risky crops. In this way, they have shown that it is necessary for governments to subsidize crop insurance. These subsidies are also present in the US, where the government subsidizes farmers by about 60% [12]. This makes these insurance policies more accessible to farmers; therefore, more farmers are interested in insuring their crops. Lusk [13] examined the effect of reducing or eliminating subsidies for crop insurance in some US states. The results showed that there were benefits in some countries from the abolition of subsidies, while in other countries there would be losses among the farmers. Santeramo and Ford Ramsey [14] attempted to answer the question of whether it is justified to subsidize crop insurance in the EU (European Union). They believe that in the long term, it would be desirable to establish a crop insurance program for the entire EU, but there are several obstacles to this.

Fang et al. [3] demonstrated in their research that crop insurance allows farmers to disperse risk and adopt sustainable agricultural practices while maintaining productivity. Jha et al. [15] proposed a shift from conventional crop insurance systems to blockchain-based crop insurance, which is more efficient, accessible, and affordable. Möhring et al. [16] saw crop insurance as an opportunity to reduce the harmful effects of pesticide use. They proved in their research that insurance helps reduce pesticide use, with pesticide costs currently being reduced by 6 to 11%. In this way, crop insurance helps promote sustainable agricultural production.

Fleckenstein et al. [17] investigated how corn producers use insurance to manage external impacts. Their research showed that efforts should continue to be made to identify risks and find solutions to increase the adoption rate of insurance. Ghosh et al. [18] found it particularly difficult to determine the actual demand for crop insurance in developing countries, so they conducted an experiment to assess how subsidies could help increase the use of crop insurance. They found that farmers have a demand for insurance.

A similar situation exists in the Republic of Serbia regarding the implementation of crop insurance. Piljan et al. [19] found that were government subsidies available, more farmers in the Republic of Serbia would use crop insurance. There needs to be a social and ecological significance that drives rural development, which would also change farmers’ attitudes toward crop insurance. Njegomir and Demko-Rihter [20] also emphasize that there should be a greater influence from the government to encourage the use of crop insurance and that incentives should be offered to increase the use of this service among farmers. In addition to government incentives, there are other factors that affect farmers’ readiness to purchase this insurance. Stojanović et al. [21] proved in their research that readiness to insure crops is influenced by age, farm size, income, and the level of risk. They obtained this information based on a sample of 255 wheat and raspberry farmers in the Republic of Serbia.

As can be seen from this literature review on crop insurance, the focus of previous research has been on government subsidies and the impact of crop insurance on green agriculture. The application of MCDM methods in crop insurance research has not been widely used in previous studies. Kurdyś-Kujawska et al. [2] used the TOPSIS technique for establishing the order of preference by similarity to the ideal solution and proved that farms with the highest productivity levels have an average insurance value compared to farms with insurance values that are twice as high in those with the lowest productivity levels. Chu and Le [22] used the AHP (analytic hierarchy process) and TOPSIS methods to select packages intended for farmers to take up crop insurance. Therefore, the application of MCDM methods to the selection of insurance companies represents a scientific contribution to this research.

## 3. Defining the Problem

In mid-May 2014, the Republic of Serbia was hit by catastrophic floods due to record amounts of rainfall. Based on reports assessing the damage, the total costs of the effects amounted to around EUR 1.7 billion, or 2.7% of the gross domestic product, in direct damage [23]. At the same time, it was estimated that around 32,500 farmers found themselves in a critical situation across the affected area, with about 80,000 hectares of arable land being flooded. In addition, a significant part of agricultural land (around 11,500 hectares) was covered with a large amount of mud and other waste material, making that part of the land unusable for a certain period of time. During this event, planted crops were destroyed, and many agricultural facilities and farm machinery were damaged. Moreover, in 2017, a drought hit the entire territory of the Republic of Serbia, causing significant damage to agriculture.

Based on this series of events, it is necessary to increase the share of crop insurance in the Republic of Serbia in order to insure against negative occurrences in agriculture. Insurance companies are introducing new policies and programs to attract new customers and retain existing ones [24]. They are offering new insurance policies that cover greater risks for farmers. However, the practice has shown that the presence of the private sector alone in agriculture produces high insurance premiums, thereby making it financially inaccessible, especially for smaller agricultural producers. Because of this, the Republic of Serbia must subsidize crop insurance in agriculture. Additionally, the key problem is selecting an insurance company that will provide the best conditions for farmers. In order to solve this decision-making problem, experts were selected for inclusion in this research. In total, five experts were selected, including three experts who are holders of agricultural holdings in the Republic of Serbia who plan to insure their crops, along with two professors from the Faculty of Agriculture and the Department of Agricultural Economics. The holders of agricultural holdings assessed which insurance company they believe provides the best conditions, while the professors evaluated these insurance companies based on their expertise in and knowledge of the economics of agriculture.

### 3.1. Defining the Alternatives

To define alternatives or insurance companies, first, it was necessary to form a basic set and then perform a causation analysis of this set. Therefore, all existing insurance companies in the Republic of Serbia were found on the website http://osiguranjeinternetom.com (accessed on 20 March 2023). A total of 16 insurance companies represent the basic set in this study. The next step was to determine which insurance companies have suitable packages for farmers. This was achieved by visiting the websites of all these insurance companies, and it was found that five insurance companies offer crop insurance services. These insurance companies are Sava (A1), Dunav (A2), DDOR (A3), Wiener (A4), and Generali (A5), and they represent the sample in this study as well as the alternatives within it. All these insurance companies have different offers for farmers, which differ in terms of insurance, insured risks, payment methods, and other insurance characteristics. Therefore, it is necessary to evaluate these insurance companies and choose the one that provides the best results when insuring crops.

Sava Insurance (A1) offers farmers the possibility of insuring their crops and fruits to reduce losses due to unforeseen circumstances. They also offer different insurance plans that would suit farmers, including selecting the type of crops, amount of goods, and insurance period. They offer insurance for grains, grapes, and certain vegetable crops. They provide this insurance based on basic and additional risks.

Dunav Insurance (A2) provides the same possibility of insuring crops and fruits, including field crops, vegetables, fruit, medicinal herbs, crops and fruits in greenhouses and glasshouses, planting materials, and fruit trees. They base their offer on basic and additional risks in agriculture.

DDOR Insurance (A3) offers insurance for all agricultural crops. It is important to note that they offer basic and additional crop insurance and have the advantage of paying only after the harvest is completed.

Wiener Insurance (A4) offers insurance for all crops, and, as with other insurance companies, they offer basic and additional insurance. They allow farmers to pay for this insurance in installments or in full as a single payment.

Generali Insurance (A5) emphasizes the fact that crop insurance has become a priority for the development of agribusiness in the Republic of Serbia. They offer insurance for field crops, vegetables, fruits, grapes, planting materials, and fruit trees. They offer their products as part of basic and additional insurance.

All these insurance companies allow farmers to use government subsidies to reduce costs for farmers. However, the cost of agricultural insurance is negligible compared to the benefits it provides to policyholders and agricultural entities. In addition, the subjective need for agricultural insurance in domestic conditions is not sufficiently developed due to low purchasing power because of the economic underdevelopment of agricultural entities, as well as low awareness of the importance of insurance [5].

### 3.2. Defining the Criteria

In order to select the insurance company that offers the best policy conditions for farmers, it is necessary to select the criteria by which these companies will be evaluated. In this study, nine criteria for evaluating the alternatives will be applied. These criteria were selected in collaboration with the experts. Together with these experts, a selection was made to cover the criteria that encompass the greatest number of characteristics of crop insurance conditions. However, not all criteria have the same importance, according to the experts. Some are more closely related to the choice, while others are less closely related. Based on this finding, the importance of these criteria is also determined. All criteria are in the form of benefit criteria, whereby if the criterion is more important, it will receive a higher rating from users. These criteria are:

The subject of insurance (C1) refers to what is insured with this type of insurance in agriculture. The subject of insurance can be a material object, property, a person, or anything else that can be injured, destroyed, or damaged [25]. In crop insurance, crops, seed material, and trees are usually insured, depending on what the insurance company covers, and the subject of insurance may vary accordingly.

The basic risk (C2) refers to the risks that an insurance company covers under the basic insurance package. It is important to choose the right package that covers the most basic risks [26], because for every additional risk that is insured, a higher insurance premium must be paid. Therefore, it is necessary to include as many risks as possible in the basic insurance package for agriculture.

The supplementary risk (C3) refers to those risks that are not covered by the basic insurance package and for which an additional insurance service must be paid. Taking on supplementary risks in insurance reduces the overall risk for farmers [27]. It is important to note that these supplementary risks may not be considered basic risks by some insurance companies. In that case, it is better to choose an insurance company that covers all these risks in the basic insurance package.

Insurance value (C4) represents the insurance premium, based on the insurance policy. It is necessary to determine the basic maximum limit of the insurance premium through the use of uncertainty theory [28]. Various mathematical models are used here to identify the maximum insurance premium while reducing risks.

Payment method (C5) represents the way in which the farmer pays for insurance, whether it is paid immediately, after the harvest, or in installments. If the insurance does not cover any possible costs that the farmer may have, he or she has no incentive to insure crops [29]. Therefore, insurance companies accommodate farmers and do not require the immediate payment of insurance but enable payment in installments or after the completed planting.

Convenience (C6) represents possible deferred interest-free payments or the subsidization of insurance by the state. It is very important to increase the use of crop insurance via the state subsidizing a certain amount of insurance [12]. This increases the use of crop insurance, and farmers are relieved of one burden of agricultural production.

Limitations (C7) represent the conditions under which farmers can take up certain insurance policies. For example, farmers must have all areas under the same culture type or have irrigation. These limitations affect the selection of insurance companies [30]. The greater the limitations, the more conditions a farmer must meet. In this case, the limitations should be as minimal as possible to make the insurance accessible to farmers.

Additional insurance (C8) refers to extra insurance that can be offered to farmers by the insurance company. Additional insurance is used by insurance companies to offer more services to farmers as part of their crop insurance. Farmers may want additional insurance to provide them with additional protection [31]. Insurance companies may offer farmers additional benefits and discounts for this additional insurance.

Insurance premium (C9) represents the monetary value of the insurance services provided by the insurance company. These are the costs that farmers agree to pay in order to have their crops protected. The insurance premium should be acceptable so that as many farmers as possible can benefit from insurance [32]. More farmers will use crop insurance if the insurance premium is more acceptable.

## 4. Proposed Methodology

Expert decision-making will be used in conducting this research. Experts will evaluate criteria and alternatives. In order to reduce the influence of experts, objective determination of weights will also be used through the fuzzy entropy method. The expert data will be in the form of linguistic assessments because the use of these assessments is closer to human thinking [33]. Therefore, fuzzy sets and methods based on this set will be used in this research—specifically, fuzzy LMAW—to determine the subjective weight of the criteria, fuzzy entropy to determine the objective weight of criteria, and fuzzy CRADIS to rank the insurance companies and select those that provide the best conditions for crop insurance in agriculture. The use of subjective and objective weights for criteria will be performed in order to reduce the influence of experts in the final decision.

### 4.1. Fuzzy LMAW Method

This method was developed by the authors Pamučar et al. [34]. Unlike other MCDM methods, this method can simultaneously determine the weights of criteria and rank alternatives [35]. However, additional steps are used for ranking alternatives with this method. In this research, only those steps used to determine the weight of the criteria will be presented.

Step 1: Prioritization of criteria. In this step, experts assess the criteria using a defined linguistic scale (Table 1). Based on this linguistic scale, experts choose the value that best corresponds to the given criterion.

Step 2: Defining the absolute fuzzy anti-ideal point (${\tilde{\gamma }}_{AIP})$. This value represents a fuzzy number that is smaller than the minimum value from the set of all priority vectors.

Step 3: Defining the fuzzy vector of the relationship $R^{e}=\left({\tilde{\eta }}_{C1}^{e},{\tilde{\eta }}_{C2}^{e},\ldots ,{\tilde{\eta }}_{Cn}^{e}\right)$, which determines the relationships between the elements of the priority vector and the absolute anti-ideal point ($\gamma_{AIP}$).

(1) $${\tilde{\mu }}_{Cn}^{e}=\left(\frac{{\tilde{\gamma }}_{Cn}^{e}}{{\tilde{\gamma }}_{AIP}}\right)=\left(\frac{\gamma_{Cn}^{\left(l\right)e}}{\gamma_{AIP}^{\left(r\right)}},\frac{\gamma_{Cn}^{\left(m\right)e}}{\gamma_{AIP}^{\left(m\right)}},\frac{\gamma_{Cn}^{\left(r\right)e}}{\gamma_{AIP}^{\left(l\right)}}\right)$$

Step 4: Determination of the vector of weight coefficients for each expert, calculated separately.

(2) $${\tilde{\omega }}_{j}^{e}=\left(\frac{\ln\left({\tilde{\mu }}_{Cn}^{e}\right)}{\ln\left(\prod_{j=1}^{n}{\tilde{\mu }}_{Cn}^{e}\right)}\right)=\left(\frac{\ln\left({\tilde{\mu }}_{Cn}^{\left(l\right)e}\right)}{\ln\left(\prod_{j=1}^{n}{\tilde{\mu }}_{Cn}^{\left(r\right)e}\right)},\frac{\ln\left({\tilde{\mu }}_{Cn}^{\left(m\right)e}\right)}{\ln\left(\prod_{j=1}^{n}{\tilde{\mu }}_{Cn}^{\left(m\right)e}\right)},\frac{\ln\left({\tilde{\mu }}_{Cn}^{\left(r\right)e}\right)}{\ln\left(\prod_{j=1}^{n}{\tilde{\mu }}_{Cn}^{\left(l\right)e}\right)}\right)$$

Step 5: Calculation of the aggregated fuzzy vectors of weight coefficients, which is performed using the Bonferroni aggregator.


(3) $$\begin{array}{cc} & {\tilde{\omega }}_{j}={\left(\frac{1}{k(k-1)}\sum_{\begin{array}{c} i,j=1\\ i\neq j \end{array}}^{k}{\tilde{\omega }}_{i}^{(e)p}{\tilde{\omega }}_{i}^{(e)q}\right)}^{\frac{1}{p+q}}=\\ & \left\{{\left(\frac{1}{k(k-1)}\sum_{\begin{array}{c} i,j=1\\ i\neq j \end{array}}^{k}{\tilde{\omega }}_{i}^{\left(l_{e}\right)p}{\tilde{\omega }}_{i}^{\left(l_{e}\right)q}\right)}^{\frac{1}{p+q}},{\left(\frac{1}{k(k-1)}\sum_{\begin{array}{c} i,j=1\\ i\neq j \end{array}}^{k}{\tilde{\omega }}_{i}^{\left(m_{e}\right)p}{\tilde{\omega }}_{i}^{\left(m_{e}\right)q}\right)}^{\frac{1}{p+q}},{\left(\frac{1}{k(k-1)}\sum_{\begin{array}{c} i,j=1\\ i\neq j \end{array}}^{k}{\tilde{\omega }}_{i}^{\left(r_{e}\right)p}{\tilde{\omega }}_{i}^{\left(r_{e}\right)q}\right)}^{\frac{1}{p+q}}\right\} \end{array}$$

Step 6. Calculation of the final values of weight coefficients. The final weights are obtained by defuzzification of the obtained aggregated fuzzy vectors of weight coefficients. In this way, the subjective weights of the criteria are obtained.

### 4.2. Fuzzy Entropy Method

The entropy method is one of the methods used for objectively determining criterion weights [36]. If the dispersion within a criterion is greater, the variance will be higher or the entropy lower, and the weight of the criterion will be greater [37]. The steps of this method are as follows.

Step 1: Formation of the initial decision matrix. The first step in any MCDM method is the formation of the initial decision matrix [38]. The same initial decision matrix is used in the application of the fuzzy entropy method and fuzzy CRADIS. This decision matrix is formed by experts evaluating the alternatives for individual criteria. To form this decision matrix, experts have evaluated these criteria for the selected alternatives using linguistic values (Table 2).

Using the membership function (Table 2), the linguistic values are transformed into fuzzy numbers.

Step 2: Normalization of the initial fuzzy decision matrix. In this step, normalization of these fuzzy values is performed. This step is basically the second step in any MCDM method. Normalization will be performed using the following formula:

(4) $$n_{ij}=\frac{x_{ij}^{l}}{\max\;x_{j}^{u}},\frac{x_{ij}^{m}}{\max\;x_{j}^{u}},\frac{x_{ij}^{u}}{\max\;x_{j}^{u}}.$$

After performing the usual steps of the MCDM methods, the steps of the entropy method are applied.

Step 3: Determination of entropy value $E_{i}.$ First, the logarithmic value of the normalized decision matrix is calculated and multiplied by the normalized data. Then, the sum of this product is formed and multiplied and is then divided by the logarithmic value of “*n*”, where “*n*” is the total number of alternatives:

(5) $$E_{i}=\frac{\sum_{j=1}^{n}p_{ij}\cdot \ln p_{ij}}{\ln n}.$$

Step 4: Calculation of the final criteria weights:

(6) $$w_{i}=\frac{1-E_{i}}{\sum_{i=1}^{m}\left(1-E_{i}\right)}.$$

These steps are used to establish the objective weight of the criteria.

### 4.3. Fuzzy CRADIS Method

The fuzzy CRADIS method represents the application of fuzzy sets according to the CRADIS method developed by Puška et al. [38]. The purpose of this method is to determine the distance between the ideal and anti-ideal values, as well as the deviation of these values in relation to the optimal values. This results in a double calculation of these deviations from the ideal and anti-ideal values and optimal alternatives. This is what differentiates the CRADIS method from other methods because they only use the deviation from a single specified criterion. The steps of the fuzzy CRADIS method are as follows [39]:

Step 1: Formation of the initial decision matrix. This step is explained above for the fuzzy entropy method.

Step 2: Normalization of the decision matrix. The same expression is used as that used above for the fuzzy entropy method.

Step 3: Creation of weighted decision matrices:

(7) $${\tilde{v}}_{ij}=\left(v_{ij}^{l},v_{ij}^{m},v_{ij}^{u}\right)={\tilde{n}}_{j}\times {\tilde{w}}_{j}.$$

Step 4: Determination of the ideal and anti-ideal values. The ideal value is the maximum value in the weighted decision matrix for individual fuzzy numbers, while the anti-ideal value is the minimum value in the weighted decision matrix for individual fuzzy numbers.

(8) $$t_{i}=\max{\tilde{v}}_{ij},\;\mathrm{gdje}\;\mathrm{je}\;{\tilde{v}}_{ij}=\left(v_{ij}^{l},v_{ij}^{m},v_{ij}^{u}\right)$$

(9) $$t_{ai}=\min{\tilde{v}}_{ij},\;\mathrm{gdje}\;\mathrm{je}\;{\tilde{v}}_{ij}=\left(v_{ij}^{l},v_{ij}^{m},v_{ij}^{u}\right)$$

Step 5: Calculation of the deviation from ideal and anti-ideal values. In this step, care is taken that this deviation is not negative so that when deviating from the ideal value, this is subtracted from that value, while in the case of deviation from the value of the weighted decision matrix, the anti-ideal value is subtracted.

(10) $$d^{+}=t_{i}-{\tilde{v}}_{ij}$$

(11) $$d^{-}={\tilde{v}}_{ij}-t_{ai}$$

At this stage, two decision matrices are formed, namely, the deviation from the ideal matrix and the deviation from the anti-ideal matrix.

Step 6: Formation of ideal and anti-ideal optimal alternatives in relation to deviations from ideal and anti-ideal values. The optimal alternative in the deviation from the ideal value matrix is the one where the deviations of individual alternatives for individual criteria are minimal. The optimal alternative in the deviation from the anti-ideal value matrix is the one that deviates the most strongly from the individual alternatives for individual criteria.

Step 7: Calculation of the sum of the deviations of individual alternatives from the ideal and anti-ideal values.

(12) $$s_{i}^{+}=\sum_{j=1}^{n}d^{+}$$

(13) $$s_{i}^{-}=\sum_{j=1}^{n}d^{-}$$

The sum of the deviations is also calculated for the optimal alternatives.

Step 8: Defuzzification of the ratings of the deviations of alternatives from ideal and anti-ideal solutions.

(14) $$s_{i}^{\pm }{}_{def}=\frac{d_{i}^{l}+4d_{i}^{m}+d_{i}^{u}}{6}$$

Step 9: Calculation of the utility function for each alternative in relation to the deviations from optimal alternatives:

(15) $$K_{i}^{+}=\frac{s_{0}^{+}}{s_{i}^{+}}\;$$

(16) $$K_{i}^{-}=\frac{s_{i}^{-}}{s_{0}^{-}}$$

where $s_{0}^{+}$ is the optimal ideal alternative and $s_{0}^{-}$ is the optimal anti-ideal alternative established in step 6.

Step 10: Ranking of alternatives.

(17) $$Q_{i}=\frac{K_{i}^{+}+K_{i}^{+}}{2}$$

The best alternative is the one with the highest value and, conversely, the worst alternative is the one with the lowest value.

## 5. Research Results

When choosing an insurance company, first, it is necessary to calculate the weight of the criteria, as this is essential information for the operation of each MCDM method. It is very important to know the weight of each criterion because this gives priority to certain criteria over others, and some will have a greater influence on decision-making than others. That is why two criteria weights are used in this study: subjective and objective criteria weights. The subjective weight is determined by the experts, while the objective weight of the criteria is determined based on the initial decision matrix. In this study, the subjective weight of the criteria will be determined first. Expert opinion and the fuzzy LMAW method will be used to calculate these weights.

The first step of the fuzzy LMAW method is the expert assessment of the criteria. Experts provide an assessment based on the linguistic values (Table 1) and select the value that they consider most appropriate for a particular criterion (Table 3). Based on this initial matrix and by using the fuzzy LMAW method, the criterion weights are calculated. After the initial decision matrix, which consists of expert assessments of individual criteria (Table 3), is formed, these values are transformed into fuzzy numbers based on the membership function (Table 1). Each linguistic value is assigned a corresponding fuzzy number.

The next step in the fuzzy LMAW method is to define the absolute fuzzy anti-ideal point. In this study, a value of 1.4 was chosen for the absolute fuzzy anti-ideal point, as it is smaller than the smallest fuzzy number. The next step is to define a fuzzy relationship vector, whereby fuzzy values are divided by the value of the absolute fuzzy anti-ideal point. The natural logarithm values are then calculated and divided by the product of the natural logarithm for each expert opinion. This process calculates the vector of weighting coefficients. The next step is to use the Bonferroni aggregator to calculate the fuzzy weights of the criteria. Finally, defuzzification is performed and the final criterion weights are calculated (Table 4).

The results of applying the fuzzy LMAW method show that the criterion with the highest weight is C9—Insurance Premium, while the criterion with the lowest weight is C8—Additional Insurance. Considering all these weights, it can be concluded that there are not too many deviations between them, and, according to the experts’ opinions, all criteria are important in making the final decision. The highest weight compared to the lowest weight is 60.77%.

After calculating the weights based on expert opinions, the weights are then calculated using the fuzzy entropy method, which is a method for objectively calculating the criterion weights. As with the fuzzy LMAW method, the first step of the fuzzy entropy method is to form an initial decision matrix. It should be noted that this initial decision matrix is the same as that used in the fuzzy CRADIS method. This decision matrix is formed by the experts giving ratings to the alternatives, based on the observed criteria. Here, experts evaluate the alternatives using linguistic values (Table 2); this value scale differs from the value scale used in the fuzzy LMAW method. This value scale evaluates the ratings from “very bad” to “very good,” with a total of seven levels (Table 5).

Afterward, these values are transformed using the membership function into the corresponding fuzzy numbers, and data normalization is performed. These two steps are the same for both the fuzzy entropy and fuzzy CRADIS methods (Table 6).

After these steps are completed, the specific steps of the fuzzy entropy method are taken. Firstly, the natural logarithm is calculated from the normalized decision matrix for all data. Then, the product of the normalized decision matrix with the logarithmic decision matrix is calculated, followed by calculating the sum of the individual criteria. After that, this sum is multiplied by the negative value of a constant (Table 7). Then, the degree of divergence is calculated, and finally, the weight of the criteria is identified. In the end, defuzzification is performed for the individual criteria. Based on this method, criterion C1—Subject of Insurance received the highest weight, while criterion C6—Convenience was the least valued. In this way, different weights for the criteria were obtained compared to the subjective and objective determination of weights.

Table 7 shows the calculation of criteria weights using the fuzzy entropy method.

To obtain the final weight of the criteria, the weights obtained by the fuzzy LMAW and fuzzy entropy methods are multiplied. This is performed using the following expression:

(18) $$w_{j}=\frac{{w^{\prime }}_{j}\cdot {w^{\prime \prime }}_{j}}{\sum_{i=1}^{n}{w^{\prime }}_{j}\cdot {w^{\prime \prime }}_{j}}.$$

Based on this expression, criterion C9—Insurance Premium received the highest weight, while criterion C3—Supplementary Risk received the lowest weight. These weights were used for ranking the alternatives.

After the final weights were calculated, the fuzzy CRADIS method was used to determine the ranking order of the alternatives. In this method, after the first two common steps, the normalized decision matrix is weighted. This is achieved by multiplying the data from the normalized decision matrix with the corresponding criterion weight. The next step is to determine the ideal and anti-ideal values. These are the maximum and minimum values in the weighted decision matrix. Then, the deviations from these values are determined, and optimal alternatives are formed. The next step is to calculate the sum of deviations for the alternatives, followed by defuzzification and the calculation of utility functions. Finally, the ranking order of the alternatives is established (Table 8). Based on the results obtained by applying the fuzzy CRADIS method, it was established that the insurance company DDOR Insurance offers the best benefits for crop insurance to farmers, while Sava Insurance showed the worst results. In order to confirm these results, a validation of the results was conducted.

Table 8 shows the deviation of alternatives from the ideal solutions and the final ranking order.

Validation of the results was performed using the same decision matrix and the same criterion weights, but the ranking order was established using different fuzzy methods [40,41,42,43]. In this study, the following fuzzy methods were used: fuzzy MARCOS (measurement of alternatives and ranking according to a compromise solution), fuzzy WASPAS (weighted aggregates’ sum product assessment), fuzzy SAW (simple additive weighting), fuzzy MABAC (multi-attributive border approximation area comparison), fuzzy ARAS (additive ratio assessment), and fuzzy TOPSIS. Each of these methods has its specific steps and ranks the alternatives in a different way. This is one of the reasons why this analysis has been performed. By using different fuzzy methods, it is possible to refute the results and prove that fuzzy CRADIS is not an adequate method for solving this research problem [44].

**Figure 1:**
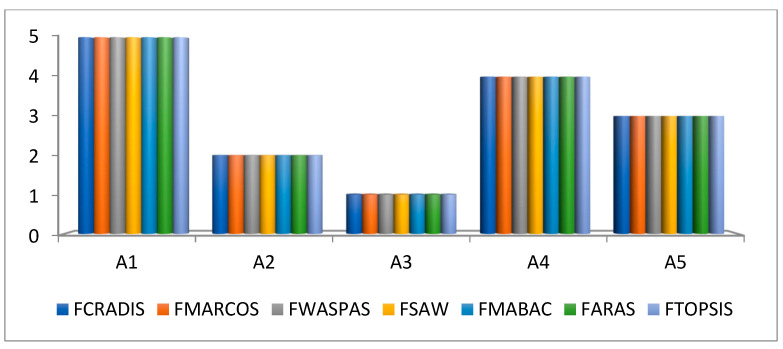
Validation of the research results.

The results of the validation show that all methods gave the same ranking order (Figure 1). In this way, the results obtained by the fuzzy CRADIS method are confirmed. After the validation of the results, further analysis was conducted to examine the influence of each criterion on the ranking order of the alternatives. This was achieved using sensitivity analysis.

**Figure 2:**
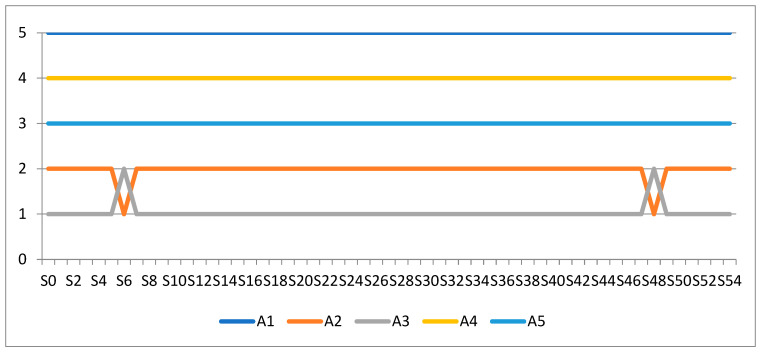
Sensitivity analysis results.

To conduct a sensitivity analysis in this study, the weight of each criterion was reduced by 15%. In this way, the weight of each criterion was reduced to 85, 70, 55, 40, 25, and 10% of its initial weight. This created a total of 54 scenarios for conducting sensitivity analysis. By conducting a sensitivity analysis, it was determined that there was little change in the ranking order of the alternatives (Figure 2). The change occurred in two scenarios, wherein the alternatives A2 and A3 swapped places. These results show that alternative A3 demonstrated better indicators for criteria C1 and C8 compared to alternative A2. Reducing the importance of these criteria showed that alternative A2 was more highly ranked than A3. Therefore, in order for Dunav Insurance to be the first choice among farmers, it must improve the subject of its insurance and offer a better choice than other insurance companies. In this way, the company would be the first choice for crop insurance among farmers in the Republic of Serbia. DDOR Insurance must improve other criteria to respond to possible moves by Dunav Insurance. By applying sensitivity analysis, the importance of individual criteria in making the final decision was further explored. However, with changes to the weights of the criteria, it was shown that there was no significant change in the ranking order of insurance companies, and, in most scenarios, the same ranking order was maintained.

## 6. Discussion

To reduce the risks found in agriculture, crop insurance is taken out. However, in order to do this, it is necessary for farmers to first understand the importance of crop insurance. This is achieved through farmer education [45]. This insurance can help farmers apply green agriculture, as there is no need to use various preparations [3]. In addition, the government must also encourage the greater use of insurance in agriculture through subsidies. Insurance companies in the Republic of Serbia cooperate with state institutions and provide certain subsidies in order to promote the use of this form of insurance among farmers. Furthermore, they offer their users various payment benefits when contracting. However, crop insurance is still not being taken up to a sufficient extent in the Republic of Serbia [21].

This study examines the problem of crop insurance in a different way from the approaches used in similar studies. Expert opinions were used to select the insurance company that provided the best conditions for farmers. For this purpose, five experts were selected, two of whom were scientific workers in the field of agriculture and three of which were farmers in the Republic of Serbia. These selected experts first evaluated the criteria of importance for them and for the insurance companies themselves. Linguistic ratings were used that are better aligned with human thinking [33]. A nine-level value scale was used to assess the importance of the criteria, while a seven-level value scale was used to assess the insurance companies. Therefore, this study used a fuzzy set.

During the formation of the initial decision matrix, which served as the basis for all decision-making processes, the criteria for evaluating insurance companies were first determined in collaboration with the experts. They identified nine key criteria that could help in making the final decision. They considered the subject of insurance, risks, insurance values and prices, and payment methods, as well as the benefits, limitations, and additional services provided by these insurance companies. In this way, they evaluated the offers of these insurance companies and provided their ratings. These ratings were transformed using fuzzy sets and were applied to the fuzzy versions of multiple criteria decision-making (MCDM) methods. Thus, information about the offers of insurance companies was provided through expert evaluations. To select an insurance company, experts also assessed the importance of individual criteria. Additionally, a cross-validation of each criterion’s importance was performed using subjective and objective ratings of the criteria weights. Furthermore, the results were validated using six different fuzzy methods.

To reduce the impact of expert judgment in determining the criterion weights, a combination of the subjective and objective determination of criterion weights was used [46,47]. Fuzzy LMAW and entropy methods were used for this purpose. The fuzzy LMAW method was chosen because it facilitates criterion evaluation. The experts did not need to rank the criteria in order of importance, but, rather, evaluated them [48], unlike other methods for determining criterion weights (FUCOM, SWARA, etc.). Based on expert evaluations made using this method, the results showed that criterion C9—Insurance Premium received the highest importance and weight. This is because insurance prices affect the overall costs of agriculture [49]. Therefore, it is necessary to choose an insurance company that will not increase the farmers’ costs. Additionally, these results showed that criterion C8—Additional Insurance was given the lowest importance. Farmers do not have to choose these insurance options because they do not affect crop insurance but rather provide additional insurance for the farmer’s machinery, facilities, etc. In this way, the farmer wants to ensure additional security by taking additional services from insurance companies [31]. The reason for this is climate change [50], which is increasingly affecting agricultural production. Based on these reasons, future research should consider using an adaptive network-based fuzzy inference system (ANFIS) since algorithms applying this system have proven to be efficient in decision support [51]. Additionally, this model reduces the possible decision-making errors made by experts.

Using the fuzzy entropy method, which was used for objectively determining the criterion weights, the results showed that criterion C1—the Subject of Insurance received the highest weight, while criterion C6—Convenience received the lowest weight. Unlike subjective methods for determining weights, objective methods determine the weights based on data dispersion within a certain criterion [52,53]. The greater the dispersion, the greater the importance of that criterion, and vice versa. These results were obtained based on this dispersion. Based on the difference between the subjective and objective methods for determining criterion weights used in this study, it was decided to choose two methods and to form criterion weights equally, based on both methods. In this way, the differences between these two approaches were considered; in this research, weights were obtained that represented a compromise between these two approaches. The reason for this can be found in the fact that the complex criteria used received less weight compared to simple criteria such as the price or object, but these complex criteria also need to be considered. It is difficult to determine this fact through a subjective approach, which is why an objective approach was used to determine the weights of the criteria.

The aim of this research was to evaluate the offerings of insurance companies in terms of crop insurance. The fuzzy CRADIS method was used to rank these insurance companies according to selected criteria and expert ratings. The results of applying the fuzzy CRADIS method showed that according to the expert ratings, the insurance company DDOR provides the best conditions for farmers in terms of crop insurance. These results were confirmed by validating the results using six other fuzzy methods. Based on this validation, this insurance company represents the first choice for farmers in the Republic of Serbia in terms of crop insurance. In the sensitivity analysis conducted, it was shown that in 2 out of 54 scenarios [54], the insurance company Dunav Insurance performed better. This is because DDOR had better indicators for criteria C1 and C8, compared to Dunav Insurance. Based on all these findings, this research has shown how fuzzy methods can be used for these and similar problems related to agricultural insurance.

In this paper, the fuzzy set methodology was used to bring decision-making closer to human thinking. With the input of selected experts, an initial decision matrix was formed, which served as the basis for making the final decision. A sensitivity analysis was conducted, revealing that the significance of individual criteria is crucial for making the ultimate decision. Therefore, it is necessary to reach a consensus, particularly in group decision-making, regarding the most important criteria for each specific problem, in order to make the final decision based on the results. Hence, future research needs to develop new models that will facilitate the process of making final decisions and, most importantly, assist farmers in improving their future production. This will ultimately enhance primary production within a country.

## 7. Conclusions

This research was conducted to determine which insurance company offers the best conditions for crop insurance in Serbia. Initially, a basic set was formed that included all insurance companies in Serbia, then those that offer crop insurance services were selected. Expert opinions and linguistic evaluations were used to assess these companies and fuzzy methods were employed in the research. A combination of objective and subjective methods was used to determine the weights of the criteria to ensure that the results were as realistic as possible. The fuzzy LMAW method was used to subjectively determine the weights of the criteria using expert evaluations, while the fuzzy entropy method was used to objectively determine the weights of the criteria. This reduced the influence of the experts on the final evaluation of the insurance companies.

The selection of the insurance company that provides the best indicators, according to expert opinion in Serbia, was carried out using the fuzzy CRADIS method. This is a newer MCDM method that was validated and found to be consistent with other fuzzy methods. The results of this method and other fuzzy methods showed that the best results were obtained by the insurance company DDOR. Sensitivity analysis showed that in 2 out of 54 scenarios, the insurance company Dunav Insurance showed better results.

The limitations of this study are mainly related to the selection of experts. However, professors from the Faculty of Agriculture and the users of these insurance companies were included in the selection of experts. In this way, the academic community and real users of this insurance were included. In future research, it will be possible to include other types of insurance related to agriculture to determine if the same order of insurance companies applies to other types of agricultural insurance.

## Figures and Tables

**Table 1:** Linguistic scale for determining the weight of criteria.

Linguistic Value	Abbreviation	Fuzzy Number		
Absolute low	AL	1	1	1
Very low	VL	1	1.5	2
Low	L	1.5	2	2.5
Medium-low	ML	2	2.5	3
Equal	E	2.5	3	3.5
Medium-high	MH	3	3.5	4
High	H	3.5	4	4.5
Very high	VH	4	4.5	5
Absolute high	AH	4.5	5	5

**Table 2:** Linguistic values for alternative evaluation.

Linguistic Variable for Alternative	Abbreviation	Fuzzy Numbers		
Very bad	VB	0	0	1
Bad	B	0	1	3
Medium—bad	MB	1	3	5
Medium	M	3	5	7
Medium—good	MG	5	7	9
Good	G	7	9	10
Very good	VG	9	10	10

**Table 3:** Initial decision matrix for calculating criterion weights using the fuzzy LMAW method.

	C1	C2	C3	C4	C5	C6	C7	C8	C9
Expert 1	E	ML	E	MH	H	H	EH	ML	AH
Expert 2	H	MH	MH	MH	H	MH	E	E	H
Expert 3	EH	E	E	H	EH	MH	H	E	AH
Expert 4	H	H	E	ML	MH	H	E	E	AH
Expert 5	H	MH	ML	E	AH	H	E	MH	AH

**Table 4:** Calculation of criterion weights using the fuzzy LMAW method.

Fuzzy rating values																
	C1			C2			C3			…	C9					
Expert 1	2.50	3.00	3.50	2.00	2.50	3.00	2.50	3.00	3.50	…	4.50	5.00	5.00			
Expert 2	3.50	4.00	4.50	3.00	3.50	4.00	3.00	3.50	4.00	…	3.50	4.00	4.50			
Expert 3	4.00	4.50	5.00	2.50	3.00	3.50	2.50	3.00	3.50	…	4.50	5.00	5.00			
Expert 4	3.50	4.00	4.50	3.50	4.00	4.50	2.50	3.00	3.50	…	4.50	5.00	5.00			
Expert 5	3.50	4.00	4.50	3.00	3.50	4.00	2.00	2.50	3.00	…	4.50	5.00	5.00			
Values of the fuzzy relationship vector																
	C1			C2			C3			…	C9			Product		
Expert 1	1.79	2.14	2.50	1.43	1.79	2.14	1.79	2.14	2.50	…	3.21	3.57	3.57	800.42	3430.37	10,805.66
Expert 2	2.50	2.86	3.21	2.14	2.50	2.86	2.14	2.50	2.86	…	2.50	2.86	3.21	1050.55	4183.52	13,831.24
Expert 3	2.86	3.21	3.57	1.79	2.14	2.50	1.79	2.14	2.50	…	3.21	3.57	3.57	2001.05	7409.60	21,011.01
Expert 4	2.50	2.86	3.21	2.50	2.86	3.21	1.79	2.14	2.50	…	3.21	3.57	3.57	875.46	3659.06	11,345.94
Expert 5	2.50	2.86	3.21	2.14	2.50	2.86	1.43	1.79	2.14	…	3.21	3.57	3.57	1350.71	5336.13	14,407.55
Values of the weight coefficient vector																
	C1			C2			C3			…	C9					
Expert 1	0.06	0.09	0.14	0.04	0.07	0.11	0.06	0.09	0.14	…	0.13	0.16	0.19			
Expert 2	0.10	0.13	0.17	0.08	0.11	0.15	0.08	0.11	0.15	…	0.10	0.13	0.17			
Expert 3	0.11	0.13	0.17	0.06	0.09	0.12	0.06	0.09	0.12	…	0.12	0.14	0.17			
Expert 4	0.10	0.13	0.17	0.10	0.13	0.17	0.06	0.09	0.14	…	0.13	0.16	0.19			
Expert 5	0.10	0.12	0.16	0.08	0.11	0.15	0.04	0.07	0.11	…	0.12	0.15	0.18			
Final weights of the criteria																
	C1	C2	C3		C4	C5	C6		C7		C8	C9				
*w″*	0.1221	0.1016	0.0913		0.1013	0.1308	0.1204		0.1083		0.0910	0.1463				

**Table 5:** Linguistic initial decision matrix.

Expert 1	C1	C2	C3	C4	C5	C6	C7	C8	C9
A1	M	MG	MB	M	MG	M	MB	M	MG
A2	M	MB	B	B	MB	VB	MB	B	B
A3	MB	M	B	MG	MB	B	MB	MB	M
A4	M	MB	B	MG	M	B	M	MB	M
A5	M	MB	MB	M	M	B	MB	MB	MB
Expert 2	C1	C2	C3	C4	C5	C6	C7	C8	C9
A1	MG	MG	M	MB	G	MB	M	MB	G
A2	M	B	VB	B	B	B	MB	MB	MB
A3	B	MB	B	M	B	MB	MB	B	MB
A4	MB	MB	B	M	MB	MB	MG	M	MG
A5	MG	M	B	M	MB	VB	B	M	MB
Expert 3	C1	C2	C3	C4	C5	C6	C7	C8	C9
A1	M	M	MB	M	M	M	MG	M	MG
A2	MB	MB	VB	VB	MB	VB	B	MB	M
A3	VB	B	MB	B	B	B	B	VB	B
A4	MB	M	MB	M	M	B	MB	MB	MG
A5	M	MB	MB	B	B	VB	B	B	MB
Expert 4	C1	C2	C3	C4	C5	C6	C7	C8	C9
A1	MB	MG	B	MB	MB	MB	MB	MB	M
A2	VB	B	VB	B	MB	B	B	MB	M
A3	VB	VB	B	VB	VB	B	B	B	M
A4	B	MG	B	MB	MB	VB	B	B	MB
A5	M	M	MG	VB	VB	MB	B	B	MB
Expert 5	C1	C2	C3	C4	C5	C6	C7	C8	C9
A1	MB	MB	M	M	MB	MB	M	MG	MG
A2	B	MB	B	VB	MB	MB	MB	MG	MB
A3	B	B	B	MB	MB	VB	B	B	M
A4	MG	MB	B	MB	MB	MB	M	MG	MB
A5	MB	B	B	MB	M	B	B	MB	M

**Table 6:** Initial fuzzy decision matrix and normalized decision matrix.

Initial fuzzy decision matrix																
	C1			C2			C3			C4			…	C9		
A1	3.40	5.40	7.40	2.20	4.20	6.20	4.60	6.60	8.40	3.80	5.80	7.80	…	1.20	3.00	5.00
A2	5.40	7.20	8.60	5.80	7.80	9.40	8.20	9.60	10.00	7.80	9.40	10.00	…	4.60	6.60	8.40
A3	7.40	9.00	9.80	6.20	8.00	9.20	6.60	8.60	9.80	5.00	6.80	8.20	…	4.20	6.20	8.00
A4	4.20	6.20	8.00	3.80	5.80	7.80	6.60	8.60	9.80	3.40	5.40	7.40	…	3.00	5.00	7.00
A5	3.00	5.00	7.00	4.60	6.60	8.40	5.00	7.00	8.60	5.40	7.20	8.60	…	4.60	6.60	8.60
Normalized decision matrix																
	C1			C2			C3			C4			…	C9		
A1	0.35	0.55	0.76	0.23	0.45	0.66	0.46	0.66	0.84	0.38	0.58	0.78	…	0.14	0.35	0.58
A2	0.55	0.73	0.88	0.62	0.83	1.00	0.82	0.96	1.00	0.78	0.94	1.00	…	0.53	0.77	0.98
A3	0.76	0.92	1.00	0.66	0.85	0.98	0.66	0.86	0.98	0.50	0.68	0.82	…	0.49	0.72	0.93
A4	0.43	0.63	0.82	0.40	0.62	0.83	0.66	0.86	0.98	0.34	0.54	0.74	…	0.35	0.58	0.81
A5	0.31	0.51	0.71	0.49	0.70	0.89	0.50	0.70	0.86	0.54	0.72	0.86	…	0.53	0.77	1.00

**Table 7:** The calculation of criteria weights using the fuzzy entropy method.

	C1			C2			C3			C4			…	C9		
$E_{i}$	1.01	0.79	0.46	1.01	0.74	0.34	0.88	0.51	0.20	1.00	0.75	0.44	…	1.03	0.82	0.36
1−$E_{i}$	0.12	0.13	0.16	0.12	0.12	0.12	0.10	0.08	0.07	0.11	0.12	0.15	…	0.12	0.13	0.12
	C1		C2		C3			C4	C5	C6	C7	C8		C9		
Entropy *w′*	0.131		0.119		0.083			0.125	0.117	0.067	0.108	0.121		0.129		
	C1		C2		C3			C4	C5	C6	C7	C8		C9		
Final *w*	0.141		0.107		0.067			0.112	0.135	0.071	0.104	0.097		0.167		

**Table 8:** The deviation of alternatives from the ideal solutions and the final ranking order.

	$s^{+}$	$s^{-}\;$	*Def* $s^{+}$	$\mathrm{Def}\;s^{-}$	$K_{i}^{+}$	$K_{i}^{-}\;$	$Q_{i}$	RANK
A1	0.65 0.65 0.78	0.10 0.11 0.21	0.671	0.129	0.471	0.266	0.369	5
A2	0.34 0.36 0.54	0.40 0.41 0.45	0.386	0.414	0.820	0.856	0.838	2
A3	0.32 0.33 0.54	0.43 0.43 0.46	0.365	0.435	0.867	0.899	0.883	1
A4	0.52 0.52 0.67	0.22 0.24 0.33	0.547	0.253	0.578	0.523	0.551	4
A5	0.42 0.43 0.60	0.32 0.33 0.39	0.457	0.342	0.692	0.708	0.700	3
*A*_0_	0.26 0.28 0.50	0.48 0.48 0.49	0.316	0.483				

## Data Availability

Not applicable.
